# An Assessment of How Clinicians and Staff Members Use a Diabetes Artificial Intelligence Prediction Tool: Mixed Methods Study

**DOI:** 10.2196/45032

**Published:** 2023-05-29

**Authors:** Winston R Liaw, Yessenia Ramos Silva, Erica G Soltero, Alex Krist, Angela L Stotts

**Affiliations:** 1 Department of Health Systems and Population Health Sciences Tilman J Fertitta Family College of Medicine University of Houston Houston, TX United States; 2 Rice University Houston, TX United States; 3 USDA/ARS Children's Nutrition Research Center Department of Pediatrics Baylor College of Medicine Houston, TX United States; 4 Department of Family Medicine & Population Health Virginia Commonwealth University School of Medicine Richmond, VA United States; 5 Department of Family & Community Medicine McGovern Medical School University of Texas Health Science Center at Houston Houston, TX United States

**Keywords:** artificial intelligence, medical informatics, qualitative research, prediction tool, clinicians, diabetes, treatment, clinical decision support, decision-making, survey, interview, usefulness, implementation, validation, design, usability

## Abstract

**Background:**

Nearly one-third of patients with diabetes are poorly controlled (hemoglobin A_1c_≥9%). Identifying at-risk individuals and providing them with effective treatment is an important strategy for preventing poor control.

**Objective:**

This study aims to assess how clinicians and staff members would use a clinical decision support tool based on artificial intelligence (AI) and identify factors that affect adoption.

**Methods:**

This was a mixed methods study that combined semistructured interviews and surveys to assess the perceived usefulness and ease of use, intent to use, and factors affecting tool adoption. We recruited clinicians and staff members from practices that manage diabetes. During the interviews, participants reviewed a sample electronic health record alert and were informed that the tool uses AI to identify those at high risk for poor control. Participants discussed how they would use the tool, whether it would contribute to care, and the factors affecting its implementation. In a survey, participants reported their demographics; rank-ordered factors influencing the adoption of the tool; and reported their perception of the tool’s usefulness as well as their intent to use, ease of use, and organizational support for use. Qualitative data were analyzed using a thematic content analysis approach. We used descriptive statistics to report demographics and analyze the findings of the survey.

**Results:**

In total, 22 individuals participated in the study. Two-thirds (14/22, 63%) of respondents were physicians. Overall, 36% (8/22) of respondents worked in academic health centers, whereas 27% (6/22) of respondents worked in federally qualified health centers. The interviews identified several themes: this tool has the potential to be useful because it provides information that is not currently available and can make care more efficient and effective; clinicians and staff members were concerned about how the tool affects patient-oriented outcomes and clinical workflows; adoption of the tool is dependent on its validation, transparency, actionability, and design and could be increased with changes to the interface and usability; and implementation would require buy-in and need to be tailored to the demands and resources of clinics and communities. Survey findings supported these themes, as 77% (17/22) of participants somewhat, moderately, or strongly agreed that they would use the tool, whereas these figures were 82% (18/22) for usefulness, 82% (18/22) for ease of use, and 68% (15/22) for clinic support. The 2 highest ranked factors affecting adoption were whether the tool improves health and the accuracy of the tool.

**Conclusions:**

Most participants found the tool to be easy to use and useful, although they had concerns about alert fatigue, bias, and transparency. These data will be used to enhance the design of an AI tool.

## Introduction

### Background

Poor control, defined as a hemoglobin A_1c_ (HbA_1c_) level >9.0%, contributes to complications, including nephropathy [1-6], retinopathy [4,7], and neuropathy [4,8]. Reducing poor control is important because a 2% decrease in HbA_1c_ (eg, from 9% to 7%) lowers the probability of microvascular complications by 50% to 76% [9]. The number of Americans with poorly controlled diabetes has been increasing, contributing to preventable morbidity and mortality [10-12]. In federally qualified health centers (FQHCs), the percentage with poor control was 32% in 2016 (up from 29% in 2009), suggesting that a new approach to diabetes management is needed [13,14]. Owing to the importance of poor control, the metric has been included in Healthy People 2030, which sets the national target at 11.6%, and in the measure sets that payers use to assess quality [15,16]. Thus, successfully reaching targets for diabetes control is important not only for patient health but also for the viability of health care organizations.

To meet these goals, researchers and clinicians are using artificial intelligence (AI) to integrate electronic health records (EHRs) and social risk factors, such as neighborhood characteristics, to predict outcomes important to individuals with diabetes, including poor control [17-22]. For instance, communities with poor housing, transportation, poverty, and education have higher rates of diabetes [23-25]. With the growth of EHRs, remote patient monitoring, and geo-tracking, the amount of data available to clinicians has increased exponentially [26]. Although this digitization offers tremendous opportunities for prediction, it also risks overwhelming clinicians [27]. This is true for primary care, which influences downstream spending and is responsible for whole person care that spans organs and diseases and serves as a point of integration with public health and behavioral health [28]. As a result of these functions, primary care clinicians are particularly susceptible to burnout, and it remains to be seen whether AI can help [29,30].

Unfortunately, the implementation of AI tools for diabetes has lagged, and few tools are used in practice, limiting their impact. A systematic review identified only 51 studies involving AI implementation [31]. Of these, 6 were related to diabetes. These applications used computer vision to diagnose diabetic retinopathy from retinal images and EHR data to predict those at risk for hyperglycemia. One study examined the implementation of a tool that predicts poor glycemic control [32]. As it was not tailored to the clinic’s resources and population, only 14% (4/28) of users indicated that they would recommend the tool to others, and many users reported that the interventions were inappropriate or not useful [32]. One possibility is that the organization failed to adequately address sociotechnical issues. The sociotechnical theory posits that the implementation of technology depends on values, mindsets, and communication and is an evolutionary process best achieved by early and active engagement with frontline workers [33,34]. Taken together, these studies indicate that a greater focus on AI implementation and end-user engagement during development are needed to tailor tools to clinical resources and workflows.

### Objectives

As the absence of engagement has the potential to reduce trust and increase errors, researchers are starting to pay attention to end users [35] and are finding that usability of and satisfaction with AI tools are generally high [35-37]. Although most of these tools have targeted specialists, 1 study examined how primary care physicians use an AI tool to diagnose skin lesions [38]. Most of these studies used quantitative methods and examined tools that have already been developed [35-37]. This study is novel because it qualitatively assesses the use of a poorly controlled diabetes risk tool that has yet to be created and is based on the theory that early engagement with clinicians and staff will lead to methodological and design decisions that will support the tool’s implementation. Furthermore, it is one of the few studies to target clinicians and staff working in primary care. The objective of this study was to assess how clinicians and staff would use and modify an AI clinical decision support tool for diabetes and to identify concerns and factors that affect its adoption and implementation.

## Methods

### Study Design and Participants

This is a mixed methods study of semistructured interviews and surveys to assess the perceived usefulness and ease of use, intent to use, and factors affecting tool adoption. The inclusion criteria were individuals (clinicians and staff) working in clinics that care for diabetes, adults aged ≥18 years, and English speakers. Participants were recruited via email through the researchers’ networks.

### Interview Procedures

Interviews were conducted by a trained interviewer between June 2021 and January 2022. All interviews were in English, conducted using a web-based platform, and audio recorded. Participants were compensated US $50 upon completion of the interview and survey.

### Ethics Approval

The protocol was approved by the Institutional Review Board of the University of Houston (STUDY00002980).

### Interview Guide

A semistructured interview guide (Multimedia Appendix 1) was developed by the research team (Textbox 1). The questions were informed by the Technology Acceptance Model. This model was developed to predict individual adoption and use of new technology. It theorizes that individuals’ intention to use new technology is determined by perceived usefulness, defined as “the extent to which a person believes that using [a new technology] will enhance his or her job performance,” and perceived ease of use, defined as “the degree to which a person believes that using [a new technology] will be free of effort” [39]. The model explains approximately 40% of the variance in individuals’ intention to use a new technology and actual use [39]. During the interview, participants were asked to review a sample EHR alert and were informed that their clinic is considering the implementation of a clinical decision support tool that uses AI. This tool incorporates data from the EHR and the neighborhood in which the patient lives to predict whether the patient will have uncontrolled diabetes. The alert indicates that the fictional patient is at high risk for having an HbA_1c_ level of >9% over the next year. The tool suggests multiple actions that could reduce the risk, including sending referrals to a social worker, dietitian, or behavioral specialist, ordering an antidepressant or a diabetes medication, and scheduling visits every 3 months.

**Semistructured interview questions.**

- What would you do with the information that you reviewed in the electronic health record alert?
- How useful, if at all, is this information for managing your patients with diabetes?
- What additional information would make this electronic health record alert more useful?
- How would you want the information presented to you so that it was easy to use?
- Would you prefer to receive this information at a specific point in time, such as at the point of care?
- To whom should this information be given? Consider clinicians, staff, administrators, and patients.
- What concerns do you have about using this tool?
- What are you already doing to identify people who are at high risk for uncontrolled diabetes?
- Besides uncontrolled diabetes, are there other undesirable outcomes that would be important to predict to improve the health of your patients?
- What are the factors that would affect whether this tool is implemented into practice at your clinic?

### Qualitative Data Analysis

The interviews were transcribed using a web-based service (Otter [40]). A research assistant checked the transcripts for accuracy and cleaned and deidentified the transcripts when appropriate. The transcripts were coded by 2 individuals using thematic content analysis in NVivo (QSR International). First, the coders read each transcript independently. On the basis of the study objectives, interview guide, and responses, codes were generated using repeated ideas. Following the first reading, the coders compared the codes and developed a guiding codebook (version 1) with a list of codes and definitions. Using the updated codebook, the coders independently applied codes to the interviews in a second reading and met to reconcile coding discrepancies and modify the codebook (version 2). The coders used the resultant codebook to conduct a final review of the interviews, coming together to reconcile differences. Coding stopped once study objectives were saturated, indicating that no new information was identified. Following the coding process, codes were organized into themes and findings. To describe the strength of ideas, we calculated the number of respondents contributing to each finding.

### Survey Design

Following the interview, the participants completed a survey (Multimedia Appendix 2). On a 7-point Likert scale (strongly disagree to strongly agree), participants reported their intent to use the tool, perceived usefulness, ease of use, and organizational support for use. Next, they rank ordered the factors influencing the tool’s implementation (cost of the tool, accuracy, health improvement, cost to the system, usability, impact on clinical workflows, and other). To quantify the extent to which AI would need to outperform clinician intuition for adoption, we asked participants to respond to the following prompt:

A team of clinicians and staff were tasked with predicting whether the 1000 individuals with diabetes in your practice would have a hemoglobin A_1c_ > 9% in the next year. The following year, your practice announced that the team accurately predicted the fate of 800 of these individuals. How many people would the AI tool need to accurately categorize for you to consider using it?

We collected demographic information, including age, gender, race and ethnicity, professional role, and practice setting. Physicians also reported the years since residency graduation and their specialty.

### Quantitative Data Analysis

We used descriptive statistics to quantify demographics and responses.

## Results

### Overview

In total, 22 individuals participated in this study. They were predominantly women, Hispanic, and physicians (Table 1). The sample also included a nurse practitioner, physician assistant, behavioral therapist, and social worker. Overall, attitudes toward the tool were favorable (Table 2). Of 22 participants, 17 (77%) somewhat, moderately, or strongly agreed that they would use the tool, whereas this figure was 18 (82%) for its usefulness. These figures were 82% (18/22) and 68%(15/22) for ease of use and clinic support, respectively. When asked to rank order the factors affecting implementation, the top 3 items were whether the tool improved health, accuracy, and usability. Finally, we asked participants to quantify how accurate the tool would need to be for them to consider using it. Of 1000 individuals with diabetes, the mean number of people whose prognosis the tool would need to accurately predict was 617 (SD 264), although the responses ranged from 20 to 900.

**Table 1:** Participant demographics (n=22).

Characteristics			Values, n (%)
**Gender**			
	Women	13 (59)	
	Men	8 (36)	
	Prefer not to answer	1 (5)	
**Race and ethnicity (select all that apply)**			
	Hispanic, Latinx, or Spanish origin	9 (41)	
	White	6 (27)	
	Asian	4 (18)	
	Black or African American	1 (5)	
	Middle Eastern or North African	1 (5)	
	Prefer not to answer	1 (5)	
**Professional role**			
	Physician	14 (64)	
	Nurse practitioner	1 (5)	
	Physician assistant	1 (5)	
	Nurse	1 (5)	
	Behavioral specialist	1 (5)	
	Social worker	1 (5)	
	Other (front desk, administrative, or medical assistant)	3 (14)	
**Primary practice site**			
	Academic health center or faculty practice	8 (36)	
	Federally qualified health center or look-alike	6 (27)	
	Private solo or group practice	4 (18)	
	Health maintenance organization (eg, Kaiser Permanente)	2 (9)	
	Mental health center	1 (5)	
	Other (multiple sites)	1 (5)	
**Specialty (includes physicians, nurse practitioners, and physician assistants)**			
	Family medicine	15 (94)	
	Pediatrics	1 (6)	
**Years since residency graduation (physicians only)**			
	In residency	4 (29)	
	1-10	4 (29)	
	11-20	3 (21)	
	21-30	3 (21)	

**Table 2:** Attitudes toward the tool and factors affecting implementation.

						Values
**Attitudes^a^, mean (SD)**						1-7^b^
	“I would use the clinical decision support tool^c^.”				5.6 (1.4)	
	“I find the clinical decision support tool to be useful in my job.”				5.7 (1.3)	
	“I find the clinical decision support tool to be easy to use.”				5.8 (1.2)	
	“In general, the clinic would support my use of this clinical decision support tool.”				5.0 (1.7)	
**Factors affecting implementation (rank order)^d^**						
	**Factor, mean (SD)**					
		Whether its use improves health			2.5 (1.7)	
		Accuracy			2.7 (1.7)	
		Usability			3.7 (1.5)	
		Impact on clinic workflows			3.9 (1.6)	
		Cost			4.2 (1.7)	
		Whether its use reduces costs to the health care system			4.3 (1.6)	
	**A team of clinicians and staff were tasked with predicting whether the 1000 individuals with diabetes in your practice would have a hemoglobin A_1c_ >9% in the next year. The following year, your practice announced that the team accurately predicted the fate of 800 of these individuals. How many people would the AI^e^ tool need to accurately categorize for you to consider using it?**					
		Values, mean (SD); range			617 (273); 20-900	
		**Distribution of responses, n (%)**				
			0-200	3 (14)		
			201-400	1 (5)		
			401-600	6 (27)		
			601-800	6 (27)		
			801-1000	6 (27)		

^a^1 indicates strongly disagrees, and 7 indicates strongly agree.

^b^Range of possible responses.

^c^n=21.

^d^1 indicates the most important factor, and 6 indicates the least important factor.

^e^AI: artificial intelligence.

Multiple themes related to care delivery and concerns about the tool’s use, adoption, and implementation emerged from the interviews (Table 3).

**Table 3:** Identified themes and subthemes (n=22).

Themes and subthemes				Participants, n (%)
**How could the tool affect the delivery of care?**				
	**This tool has the potential to be useful because it provides information that is not currently available and can make care more efficient and effective**			
		The tool is not currently available, addresses a clinical gap, and represents a departure from the status quo.	7 (32)	
		Clinicians and staff would increase their focus on diabetes, by scheduling more frequent visits, interacting with patients in between visits, managing diabetes even when acute issues emerge, and providing targeted education.	20 (91)	
		This tool could improve population health, address quality measures, and contribute to efficient resource allocation.	10 (45)	
		The tool would facilitate individualized and holistic care, by integrating primary care, behavioral health, and social care.	11 (50)	
		Participants were ambivalent about the tool’s impact on populations that have been made susceptible. Some participants thought these were the patients who needed attention the most, whereas others thought that making a positive impact would be difficult.	7 (32)	
**What concerns do clinicians and staff have about the tool?**				
	**Clinicians and staff were concerned about how the tool affects patient-oriented outcomes and clinic workflows**			
		Participants were concerned the tool would lead to harms, contribute to overdiagnosis, be used punitively, and make care more expensive.	15 (68)	
		The utility is limited for those clinicians who know their patients well or have access to existing programs, and some would rather focus on people who are already uncontrolled.	8 (36)	
		Participants were concerned that the tool would exacerbate existing problems, such as health disparities and alert fatigue.	14 (64)	
		Participants were concerned that the tool’s accuracy and implementation were not supported by evidence.	5 (23)	
**What changes would increase adoption?**				
	**Adoption of the tool is dependent on its validation, transparency, actionability, and design and could be increased with changes to the interface and usability**			
		The tool needs to be validated against patient-oriented outcomes so that clinics can quantify the potential return on their investment.	4 (18)	
		Knowing how the tool was developed and the rationale behind why an individual is high risk allows clinicians and staff to gauge the tool’s credibility.	11 (50)	
		To act on the information, clinicians and staff need to understand which risk factors are modifiable and which actions will have the greatest impact on lowering risk.	6 (27)	
		Using user-centered design principles has the potential to minimize the tool’s impact on workflows and maximize readability.	13 (59)	
		The ability to customize the tool is important because implementation could differ across practices and clinicians.	2 (9)	
		Participants recommended integrating functionality and relevant information from within the EHR^a^.	19 (86)	
		Participants recommended other events that could be predicted, including cardiovascular disease, uncontrolled hypertension, worsening depression, care gaps (eg, preventive services), and missed appointments.	22 (100)	
**What factors would affect implementation?**				
	**Implementation would require buy-in and need to be tailored to the demands and resources of clinics and communities**			
		The local context affects what can be done in response to the information provided by the tool. Conversely, participants will become frustrated if the tool recommends an option that is not available.	12 (55)	
		Responding to the tool in a comprehensive manner requires the engagement of a comprehensive team. Although there was strong consensus regarding the role of clinicians and nurses, participants expressed ambivalence regarding administrators and patients.	21 (95)	
		Participants wanted to share this information with patients to empower them and support transparency but were also concerned that the information would cause confusion and stress.	20 (91)	
		There was a lack of consensus regarding when the alert should appear, with some wanting it at the point of care, whereas others wanted to review the information outside of visits (eg, periodic lists or a dashboard).	17 (77)	
		Successful implementation would require trialability, training, interoperability, and buy-in.	8 (36.)	

^a^EHR: electronic health record.

### Theme 1

The tool has the potential to be useful because it provides information that is not currently available and can make care more efficient and effective.

When asked about how the tool could affect care, several participants (7/22, 32%) noted that such a tool does not exist and that it would fill a gap:

No, we don’t already have a system. So I think there is value in adding a tool that would help improve care.

Physician, academic health center

...a lot of it [clinician decisions] is...individual clinician suspicion...a lot of it is going to be based on how well each clinician knows their patients.

Physician, academic health center

Other participants argued that the tool would facilitate the delivery of proactive care, building on the core function of primary care:

The primary argument for this tool...is that it’s easier to prevent something than it is to cure it.

Physician, academic health center

...the heart of what we do in primary care is to try to help patients with chronic conditions avoid long term complications of those conditions...if [AI believes] this person might be at greater risk, I might see [that patient] more often. I might spend more time with them. I might ask different questions because I would be trying to prevent [the complication].

Physician, academic health center

As a result of using the tool, clinicians and staff thought they would increase their focus on diabetes by scheduling more frequent visits, interacting with patients in between visits, managing diabetes even when acute issues emerge, and providing targeted education (20/22, 91%):

I find that for patients who are diabetic, it is the frequency of touches at every opportunity to control their diabetes that makes the biggest difference. And so if a patient has come in for a cold, or even anything else, other than diabetes, there’s an opportunity to intervene. For those patients who are poorly controlled, it’s usually because they’re engaging with a system very infrequently. And so from that perspective, getting them reengaged in the system to become familiar with a system becomes the most valuable tool.

Physician, Health Maintenance Organization

...it...makes you think twice...it...makes you pay attention a little bit closer, and makes [you] ask, okay, why are they at risk? What are the things that I can do to reduce the risk?

Physician, private solo or group practice

...awareness is probably some of the best medicine you can give. And my philosophy is empowering a patient to give them the education, so they can make better decisions moving forward...I’m trying to empower this patient to take control of their own care.

Physician, private solo or group practice

Others believed that the tool could be used to improve elements of population health, such as improving the quality of care delivered and allocating resources to high-need patients (10/22, 45%):

...as a clinician, it’s part of my responsibility to have some awareness of the...health...of...my small population...And so this would help to do some of that.

Physician, private solo or group practice

And also, it’s part of our billing, and HEDIS measures anyway, we’re supposed to have A1cs that are below eight, and so I feel like this is designed to meet that standard.

Physician, academic health center

[Knowing which patients are at high risk is] kind of helpful...[it tells you] where to put your resources.

Nurse practitioner, FQHC

By integrating information about mental health and social risk factors, our participants (11/22, 50%) believed that the tool would facilitate individualized, holistic care:

Now that [AI] has brought it up...I would explore things...that cause high A1c’s like social determinants, depression, medical intensification...

Physician, academic health center

I think it would be very useful, because it really takes a kind of a holistic approach of looking at the entire patient, and not just, I’m not just looking at like their blood sugar.

Behavioral specialist, FQHC

I would provide education about the connection between depression and diabetes, and how they can very much go hand in hand, and how a diabetes diagnosis can either lead to a depression diagnosis or exacerbate depression that’s already there.

Social worker, FQHC

Participants were ambivalent about the tool’s impact on susceptible populations. Some participants thought that these were the patients who needed attention the most, whereas others thought that making a positive impact would be difficult (7/22, 32%):

I think definitely…in [an] underserved population, it might be more beneficial, especially since they have less access to care.

Physician assistant, FQHC

Say...I have...10 patients in the morning, and all of them have this alert, and so for all of them, I’m taking...these extra steps to identify barriers...that’s going to take more of my time.

Physician, private solo or group practice

The whole predicting, based on community or...based on where the person lives...struck me a little odd...it feels almost like...an overgeneralization...[because] you come from this community, you are at risk...Are we stereotyping?...Are we making assumptions...because someone comes from...poverty, or...a certain marginalized population?

Social worker, FQHC

### Theme 2

Clinicians and staff were concerned about how the tool affects patient-oriented outcomes and clinic workflows.

Participants had myriad concerns about the tool. First, they were concerned that the tool would lead to harms, contribute to overdiagnosis, be used punitively, and make care more expensive (15/22, 68%):

Would it make care worse? Yeah, potentially...So if you’re prompted to prescribe medications...for people who are not yet at a certain level of risk, the [benefit to harm] ratio becomes smaller.

Physician, academic health center

I would be concerned about [the] over identification [and] over diagnosis.

Physician, private solo or group practice

I think that increasing the cost of care is definitely going to happen...in many systems because of how healthcare is paid for. So if I make a referral...for the patient, and the patient has to go and pay for the social worker [and] dietitian, I’ve just increased the cost of care.

Physician, academic health center

I think that it is important to not make it look like...the fact that [patients are still uncontrolled]...is [because] you [are] a bad physician...I’m tired of that.

Physician, academic health center

In particular, those clinicians who know their patients well or have access to existing programs thought the utility was limited, and some would rather focus on people who are already uncontrolled (8/22, 36%):

...a lot of it is going to be based on how well each clinician knows their patients, and how well and how comfortable the patient feels and speaking up on their own behalf for concerns that might have arisen.

Physician, academic health center

We are asked on a monthly basis to review our patients who are not at a goal hemoglobin A1c level. Our...focus in the last six months has been...around Latino patients...So I find...this particular...information to be less valuable because we’re kind of doing it on a monthly basis already.

Physician, health maintenance organization

I would probably focus on the people I know who already have A1c’s more than 9% and start working on that population first.

Physician, mental health center

They were also concerned that the tool would exacerbate existing issues such as health disparities and alert fatigue:

Racial bias is...something that’s implicitly existent in normal data sets...this is something that just compounds...It’s like a small mistake that compounds into something bigger.

Physician, private solo or group practice

...the primary concern stems from excess information being available...But if there’s already a lot of data points, and they’re not...actionable, it can be overwhelming or just ignored.

Physician, health maintenance organization

Finally, participants were concerned that the tool’s accuracy and implementation would not be supported by evidence (5/22, 23%):

If it’s things that are [inaccurate and] manually entered into the EHR system that are driving this..., it certainly could create false alerts and waste time or...miss people who actually are at risk because...things weren’t...entered correctly, or left blank.

Physician, private solo or group practice

You have to prove to me first that identifying and managing folks like this can actually help.

Physician, academic health center

It’s only useful if I trust the information.

Physician, academic health center

### Theme 3

Adoption of the tool is dependent on its validation, transparency, actionability, and design and could be increased by changing the interface and usability.

The tool needs to be validated against patient-oriented outcomes so that clinics can quantify the potential return on investment (4/22, 18%):

The factors would be how useful the tool is, first of all, how validated the tool is and if you can show that...it changes outcomes.

Physician, private solo or group practice

The participants expected a degree of transparency and wanted to know how the tool was developed and the rationale behind the high risk of an individual. This information allows them to gauge the tool’s credibility (11/22, 50%):

...if I’m going to use a tool, I want to be able to...click a link [that] will take me to the website and I can just learn more [about] where this is being trained.

Physician, private solo or group practice

It would be helpful to know why that patient is at risk. And that will make you believe it or not.

Physician, private solo or group practice

I think some sort of report that shows me which factors contributed the most to these alerts may help me even more.

Physician, academic health center

Knowing why someone is at high risk is necessary but insufficient. Participants also wanted to understand which risk factors are modifiable and which actions will have the greatest impact on lowering risk (6/22, 27%):

...if the evidence says social work drops the risk by 50% [and] dietitian...drops the risk by 40%, on average, but in my patient, the alert fired because of nutritional concerns, I might choose the dietitian as a first choice because it might have a greater impact for this patient in particular.

Physician, academic health center

...what would be really helpful...would be some sense of the potential impact of each of these, because I’m not going to be able to get my patient to do all six potentially. But if they were organized in such a way to say this step will reduce the risk by this much. That step will reduce the risk by less...then I might be able to prioritize.

Physician, academic health center

Participants believed that perceived usability and readability would be key drivers of adoption (13/22, 59%):

[Adoption] would depend very, very, very heavily on the provider perception of usefulness and usability.

Physician, academic health center

Instead of showing six [actionable steps]...you...could [show] fewer options and color [code them]...from most benefit to least benefit.

Physician, academic health center

The ability to customize the tool is important because implementation could differ across practices and clinicians (2/22, 9%):

...there’s a lot of customization that would have to occur on the front end, to make sure that these...action items are clickable [and that] applicable resources [are] available.

Physician, private solo or group practice

Participants recommended integrating functionality and relevant information within the EHR (19/22, 86%). They wanted to include a wide range of laboratories and vital signs to provide a context for risk prediction and broaden the types of actions that could be completed within the tool:

...one of the hard parts about managing diabetes is knowing...they need another agent, and then maybe which agent the insurance might cover...it would be even more beneficial if [the tool told] me these might be suggestive agents to add...for [better] control.

Physician, academic health center

I’d want to know when and what their last hemoglobin A1c was and when their last appointment was. And then I want to know if they have seen a dietitian in the past and how long ago?

Physician, mental health center

Participants thought that this model could be applied to other conditions and recommended that the tool be used to predict important events in primary care, including cardiovascular disease, uncontrolled hypertension, worsening depression, care gaps (eg, preventive services), and missed appointments (22/22, 100%):

...you could apply the same sort of thing to preventive care to any chronic disease to including depression, hypertension, coronary disease.

Physician, academic health center

...how likely is this person going to follow through on their screenings, [like] getting their mammogram?

Physician, private solo or group practice

### Theme 4

Implementation would require buy-in and need to be tailored to the demands and resources of clinics and communities.

The local context affects what can be performed in response to the information provided by the tool. Conversely, participants will become frustrated if the tool recommends an option that is not available (12/22, 55%):

[My use of the tool] would depend a great deal on what resources are actually available to me.

Physician, academic health center

...depending on...what your clinics resources are, if you’re getting alerts for people that you have no ability to help, because you don’t have access to a social worker...that doesn’t feel really good.

Physician, academic health center

Responding to the tool in a comprehensive manner requires the engagement of a comprehensive team. Although there was strong consensus regarding the role of clinicians and nurses, participants expressed ambivalence regarding administrators and patients (21/22, 95%). All members of the primary care team have potential roles to play, including front desk personnel, pharmacists, and social workers. As roles differ for each practice, the recipients of the information may be practice dependent:

...staff should have the means to be able to respond to...this...there would be a lot of a lot of value in having multiple eyes on this to make sure that nobody falls through the cracks.

Physician, in residency

I don’t think this would be terribly helpful for administrators. Sometimes it’s used punitively. And I don’t think that that’s what we want.

Physician, academic health center

Regarding who should receive this information: “I feel like each location might want to designate that.”

Physician, academic health center

Participants wanted to share this information with patients to empower them and support transparency but were also concerned that the information would cause confusion and stress (20/22, 91%). They thought that the information without context could be harmful and that they would need scripts to explain the results in a patient-centric manner:

I think [who should receive the information] would be very, very practice dependent...I think giving the information to patients can be really valuable. I think how it’s presented and how it’s framed [is important].

Physician, academic health center

I think just a lack of context for the patient on why these certain things were ordered would be [a] concern for high alert with the patient...[patients] having no clue what it means could create...panic or some distress in the patient.

Physician, in residency

There was a lack of consensus regarding when the alert should appear, with some wanting it at the point of care, whereas others wanted to review the information outside of visits (eg, periodic lists or a dashboard, 17/22, 77%):

This really depends on the operator. For me...if it comes too early, I’ll lose it...So...I feel like [the timing] should be adjustable. That would be best because every provider is very different.

Physician, private solo or group practice

Another thing would be making sure that it’s the right time. So again, if I’m in room with the patient, personally, I don’t want to see these pop up, because I’m probably goal-oriented at that moment where I’m trying to put in something specific and this would just slow me down.

Physician, academic health center

I would be more likely to address it...if it was something I was prompted with when I opened the labs specifically...I’m going there to review their hemoglobin...I’m going there to review their lipids...so if I’m going [to the chart] for that, and...I’m prompted with this, then then I’m going to be more likely to address it right at that moment.

Physician, in residency

I wouldn’t want a list of 500 patients, because there’s no way that anybody’s going to keep track of that...that would be very difficult.

Social worker, FQHC

Successful implementation would require trialability, training, interoperability, and buy-in (8/22, 36%):

I would definitely be open to trialing it but would do it in a quality improvement sort of a mindset where we saw how things were going beforehand and how things were going afterwards. And if it didn’t help me, then I wouldn’t continue using it.

Physician, private solo or group practice

Also takes education. So educating providers about what this alert is and what this means and what we what we do with it.

Social worker, FQHC

## Discussion

### Principal Findings

From the surveys, respondents found the tool to be useful and easy to use and, if available, would use it. During the interviews, they noted that the tool is not available now and would generally change their behavior. With notable exceptions, many participants reported that their organizations lacked a systematic approach for reducing the percentage of those who are poorly controlled. Despite these benefits, the tool was not uniformly accepted, with several respondents indicating that it did not provide useful information for those patients who are well known to the practice and for those practices already offering comprehensive services. Others were concerned that AI would perpetuate biases and that alert fatigue would contribute to burnout. To enhance adoption, respondents wanted to know why the patients were at risk and what could be done to reduce that risk. Finally, they wanted to be able to tailor the tool to their local environment, noting that the suggestions offered and the recipients of the information needed to be customized to the resources, needs, and workflows of their unique clinics.

Our findings align with, and build on, the work of others. For example, similar to our results, other clinicians have responded favorably to the usability of tools that use AI [36,37]. Although usability and accuracy were deemed important, our respondents asked for steps that could be taken in response to predictions and wanted to know that those actions would lead to better health, echoing the sentiment found in other studies [35]. Similar to others, they also regarded the technology with skepticism [35,41,42]. For many years, researchers and policy makers have issued warnings regarding the black-box nature of AI and its role in widening disparities [43,44]. Our findings demonstrate that these are not theoretical issues. The clinicians and staff members in our study called for greater explainability (ie, justifications for the tool’s output), wanted these issues explicitly acknowledged and addressed, and cautioned that these tools will continue to languish on shelves in the absence of satisfactory solutions [44]. They are concerned about how AI can perpetuate the racial biases embedded within data sets and about their role in supporting biased systems. Taken together, these findings highlight the importance of the tool’s actionability, explainability, and harm minimization (resulting from bias and workflow disruptions) for its implementation and provide a blueprint for researchers interested in developing AI tools for primary care settings. For example, to address these concerns, researchers must engage communities and end users early in the development process to identify and mitigate sources of bias and iteratively test and refine the tool’s impact [45].

There are several limitations to this study that should be considered when interpreting these results. First, because we recruited participants from our networks, many of them were from academic settings and FQHCs. Our results may differ if we had a sample that is more representative of primary care clinics across the United States. Second, we did not ask the participants to use a prototype of the tool when responding to the questions. If they had, their responses to the questions regarding ease of use and usefulness may have been different. However, we contend that incorporating input from end users before a prototype is created is important for adoption. Finally, we did not assess other factors that influence adoption, such as computer self-efficacy, that we did not assess.

### Conclusions

Most participants found the tool to be easy to use and useful. They also believed that the tool could improve population health and contribute to individualized care. Conversely, participants were concerned about alert fatigue, bias, and transparency. To gauge the tool’s credibility, they wanted to know why the patients were at high risk and what they could do to reduce that risk. These data will be used to inform the development of an AI tool for diabetes.

## Appendix

Multimedia Appendix 1 Interview guide.

Multimedia Appendix 2 Clinician survey.

## Abbreviations

AI: artificial intelligence
EHR: electronic health record
FQHC: federally qualified health center
HbA_1c_: hemoglobin A_1c_
