# Supplementary material for: An Assessment of How Clinicians and Staff Members Use a Diabetes Artificial Intelligence Prediction Tool: Mixed Methods Study
Source: JMIR AI. 2023 May 29;2:e45032. doi: 10.2196/45032 (PMC11041401; doi:10.2196/45032)
Supplement: Multimedia Appendix 1 [file ai_v2i1e45032_app1.docx]

**Multimedia Appendix 1.** Interview guide.

| **Question Prompt** | **Answer Choices / Follow up Questions** |
| --- | --- |
| **INTERVIEW GUIDE** |  |
| Today I wanted to spend time talking with you about a new tool that we are developing. Specifically, I want to ask you some questions that will help us understand how clinicians would use a clinical decision support tool that is based on artificial intelligence. Remember from the consent form, that any information that you share today will be kept confidential. I am going to take a few notes as we talk; however, I can’t write everything down, so I am also going to record our conversation. I will never identify you or your place of employment during the recording. Please speak clearly and do your best in answering each question. The best answers are those that are correct for you. If you are not ok in answering a certain question, you do not need to answer that question. If you need to, you can stop me and ask questions at any time. Also, we can stop or take a break if you need one. Before we begin, do you have any questions?  [Answer questions]  Ok, starting the recorder now. | |
| What is your role in your organization? |  |
| The next several questions pertain to the following scenario and electronic health record (EHR) alert.  [Share image on screen, ask patient to view image on their screen, or ask patient to print this out while answering the questions]  You are a primary care physician working in an outpatient clinic. Because of new contracts that provide incentives for reducing both costs and the number of patients with a hemoglobin A1c > 9%, your clinic is thinking about using a clinical decision support tool that integrates with your EHR and is driven by artificial intelligence (AI). This tool incorporates data from the EHR and where the patient lives (for example, the amount of poverty in their neighborhood) to predict those patients who will have a hemoglobin A1c over 9% in the next year. Studies have confirmed the accuracy of its predictions in multiple settings. An example of the EHR alert is below, take a minute to review the EHR alert:  [If asked, artificial intelligence is as an overarching term that includes machine learning algorithms and uses software to mimic human cognition in the analysis, presentation, and comprehension of health care data.]  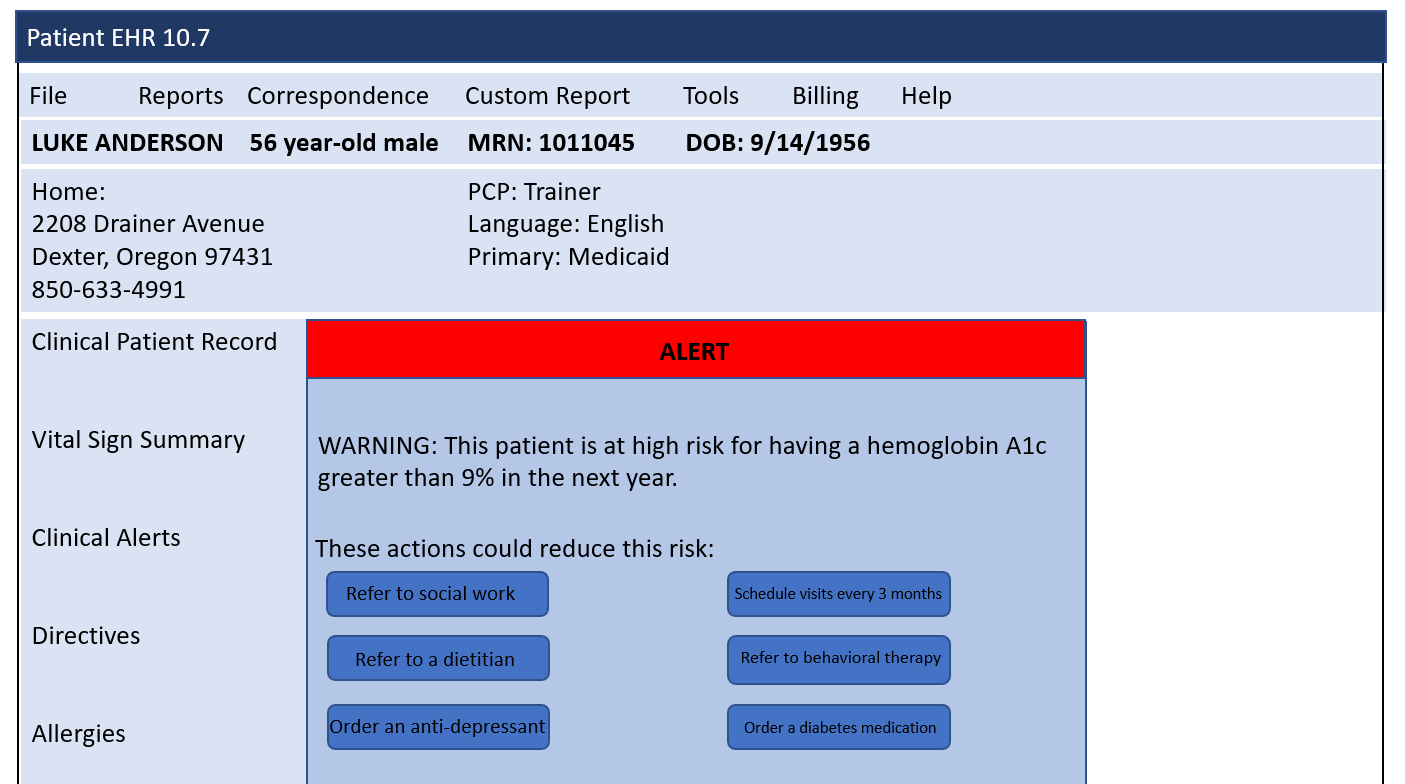 | |
| What would you do with the information that you reviewed in the EHR alert? | If needed, prompt with: Would you…?  Generate a referral to the appropriate team member (social work, dietitian, pharmacist, behavioral therapy, community health worker)  Schedule visits every 3 months  Make changes to the medication regimen (diabetes, depression)  Discuss the findings with the patient  Spend more time with the patient  Gather additional information about the patient’s social context  Nothing  Other |
| How useful, if at all, is this information for managing your patients with diabetes? | Follow up: In your opinion, why is this information useful? OR In your opinion, why is this information not useful? |
| What additional information would make this EHR alert more useful? |  |
| How would you want the information presented to you so that it was easy to use? | If needed, prompt with: Would you prefer to receive this information at the point of care, a list of high-risk patients in your panel, or both?  Why do you prefer this option? |
| To whom should this information be given? Consider clinicians, staff, administrators, and patients. | Why should these individuals receive this information?  Why should the others not receive this information? |
| What concerns do you have about using this tool? | If needed, prompt with: Are you concerned about…?  Receiving too many alerts  The accuracy of the predictions  Not knowing what to do with the information  The tool making care worse  The tool increasing the cost of care  The tool not providing additional value. In other words, you already have a good system for identifying people who will be uncontrolled. |
| What are you already doing to identify people who are at high risk for uncontrolled diabetes? | Follow up: Based on the answer given, would this tool contribute to the things that you are already doing to identify people at high risk for uncontrolled diabetes? |
| Besides uncontrolled diabetes, are there other undesirable outcomes that would be important to predict in order to improve the health of your patients? | What actions could be taken to reduce the risk of the proposed outcome? |
| What are the factors that would affect whether this tool is implemented into practice at your clinic? | Prompt: What factors would determine whether your clinic would or would not implement this tool into practice? |
| Before we end our time today, are there any other thoughts regarding this tool that you would like to share? |  |
| Ok, I’m going to turn off the recorder now. I want to thank you for your time in answering my questions. The feedback that you shared will help us to determine how clinicians would use a clinical decision support tool that uses artificial intelligence. Following this interview, you will receive a follow up survey via email. This survey should take about 10 minutes to complete. After completion of the interview, we will send you a $50 gift card via email. Do you have any questions before I end the call?  [Answer Questions]  Ok, thanks again for your time and if you have any questions in the future regarding your participation, please contact the research team at 713-348-9862. This contact information is also listed on your copy of the consent form.  [End call] | |
