# Supplementary material for: An Assessment of How Clinicians and Staff Members Use a Diabetes Artificial Intelligence Prediction Tool: Mixed Methods Study
Source: JMIR AI. 2023 May 29;2:e45032. doi: 10.2196/45032 (PMC11041401; doi:10.2196/45032)
Supplement: Multimedia Appendix 2 [file ai_v2i1e45032_app2.docx]

**Multimedia Appendix 2.** Clinician survey.

| **QUESTIONS FOR SURVEY** |  |
| --- | --- |
| First Name |  |
| Last Name |  |
| I would use the clinical decision support tool. | 1 – Strongly disagree  2 – Moderately disagree  3 – Somewhat disagree  4 - Neutral (neither disagree nor agree)  5 – Somewhat agree  6 – Moderately agree  7 – Strongly agree |
| I find the clinical decision support tool to be useful in my job. | 1 – Strongly disagree  2 – Moderately disagree  3 – Somewhat disagree  4 - Neutral (neither disagree nor agree)  5 – Somewhat agree  6 – Moderately agree  7 – Strongly agree |
| I find the clinical decision support tool to be easy to use. | 1 – Strongly disagree  2 – Moderately disagree  3 – Somewhat disagree  4 - Neutral (neither disagree nor agree)  5 – Somewhat agree  6 – Moderately agree  7 – Strongly agree |
| In general, the clinic would support my use of this clinical decision support tool. | 1 – Strongly disagree  2 – Moderately disagree  3 – Somewhat disagree  4 - Neutral (neither disagree nor agree)  5 – Somewhat agree  6 – Moderately agree  7 – Strongly agree |
| A team of clinicians and staff were tasked with predicting whether the 1000 individuals with diabetes in your practice would have a hemoglobin A1c > 9% in the next year. The following year, your practice announced that the team accurately predicted the fate of 800 of these individuals. How many people would the AI tool need to accurately categorize for you to consider using it? | Enter a number (0-1000) |
| Assume that you are the person responsible for deciding whether the tool is implemented. How would you rank these factors when making your decision? | Rank the following factors 1-6, with 1-indicating the most important factor and 6 indicating the least important factor  Cost  Accuracy  Whether its use improves health  Whether its use reduces costs to the health care system  Usability  Impact on clinic workflows |
| What additional comments do you have about using or adopting this tool? | Free text |
| What is your age? | Number |
| Which of the following best defines your gender identity? | Male  Female  Trans male / trans man  Trans female / trans woman  Genderqueer, gender non-binary, or gender non-conforming  Prefer to self-describe  Prefer to not answer |
| Which of the following best defines your race or ethnicity? Select all that apply. | American Indian or Alaskan Native  Asian  Black or African American  Hispanic, Latino/a, or Spanish origin  Middle Eastern or North African  Native Hawaiian or Other Pacific Islander  White  Prefer to self-describe  Prefer not to answer |
| How would you describe your professional role? | Physician  Nurse practitioner  Physician assistant  Nurse  Pharmacist  Behavioral specialist  Social worker  Other |
| Year of graduation from residency (if physician) |  |
| What is your specialty? | Family medicine  Internal medicine  Endocrinology (adult or pediatrics)  Pediatrics  Medicine – Pediatrics  Other |
| Which of the following describes your primary practice site ownership?  (Select one) | Private solo or group practice  Freestanding urgent care center  Hospital emergency department  Hospital outpatient department  Ambulatory surgical center  Industrial outpatient facility  Mental health center  Non-federal government clinic (e.g., state, county, city, maternal and child health, etc.)  Federally Qualified Health Center or Look-Alike  Rural Health Clinic  Indian Health Service  Institutional setting (School-based Clinic, Nursing home, prison)  Academic Health Center / Faculty Practice  Health maintenance organization (e.g., Kaiser Permanente)  Federal (Military, Veterans Administration/Department of Defense)  Public Health Service  Other ____ (Free text) |
